# Problematic smartphone use among moroccan medical students

**DOI:** 10.1192/j.eurpsy.2023.819

**Published:** 2023-07-19

**Authors:** H. Choujaa, N. Attouche, M. Agoub, K. Mchichi Alami

**Affiliations:** Psychiatrie, Chu Ibn Rochd, Casablanca, Morocco

## Abstract

**Introduction:**

While Smartphone use has been increasing all across age sectors, university students are the largest consumers group of Smartphone services. However, their excessive use or addiction can have harmful effects on the mental and physical health of their users. It is a real public health problem that is growing and affects especially the young population.

**Objectives:**

We aim to assess the prevalence of smartphone addiction among medical students in Casablanca, Morroco, describe the profile of techno-addictive students (Smartphones) and assess the risk of developing somatic and psychic conditions.

**Methods:**

This is a descriptive analytical cross-sectional study, from October 2020 to March 2021, a sample of 878 students of the Faculty of Medicine and Pharmacy of Casablanca, Morocco, including all levels of the academic year 2020/2021. This sample was calculated on the basis of the number of medical students at the Faculty of Medicine and Pharmacy of Casablanca 4095, with an expected prevalence of 51%. Data were collected anonymously through an online questionnaire, constructed of several sections including the SAS-SV scale.

**Results:**

The age of the participants ranged from 17 to 32 years, average age was 22.03 with a M/F ratio of 0.43. Students ranged from first grade to eighth grade, with a majority of Moroccan nationality97.15%. The study revealed a total percentage of addiction of 37.9% for both sexes. Psychic signs such as anxiety, loss of control, disturbance and withdrawal were more frequent than physical signs such as wrist and neck pain. After the analysis of the different results, we come out with the following conclusions concerning the profile of the participants affected by smartphone addiction: Most of them are single94.76%, with a medium socio-economic level 55.5%, with another addiction 31.2%, especially to psychoactive substances,15% with psychiatric history, 45.22%with less than 5 years of smartphone use.

**Table I:** Univariate associations of smartphone use and smartphone addiction.

CATEGORY	SUB CATEGORY	SMARTPHONE ADDICTION ACCORDING TO THE SAS-SV	NO ADDICTION	P VALUE		
		NUMBER	POURCENTAGE	NUMBER	POURCENTAGE	
Sexe	Female	137	23.1%	455	76.9%	<0,001
Nationality	Moroccan	266	31.2%	587	68.8%	0.028
Medical history	Yes	45	25.4%	132	69.1%	0.042
Psychiatric history	Yes	30	23.1	100	76.9	0.021
Taking treatment prescribed by the Psychiatrist	No	259	33%	527	67%	0.029
Substance use	Yes	116	42.3%	158	57.7%	0.001
Period of use of the smartphone	<5 years	149	37.5%	248	62.5%	0.001
Internet access	Yes	254	30.9%	556	74.6%	0.358

**Conclusions:**

In conclusion, the present study provides the first insights into smartphone use, smartphone addiction, and predictors of smartphone addiction in young people from Morocco. Future studies should extend this knowledge in order to draw clearer conclusions regarding the disease burden, and why not a more precise long-term exploration of the fate of these students and their later risk in the professional hospital setting seems worth studying.

**Disclosure of Interest:**

None Declared

